# Phenolic Compounds from Sesame Cake and Antioxidant Activity: A New Insight for Agri-Food Residues’ Significance for Sustainable Development

**DOI:** 10.3390/foods8100432

**Published:** 2019-09-22

**Authors:** Reham Hassan Mekky, Essam Abdel-Sattar, Antonio Segura-Carretero, María del Mar Contreras

**Affiliations:** 1 Department of Pharmacognosy, Faculty of Pharmacy, Egyptian Russian University, Badr City, Cairo-Suez Road, Cairo 11829, Egypt; reham-mekky@eru.edu.eg; 2 Research and Development Functional Food Centre (CIDAF), Bioregiόn Building, Health Science Technological Park, Avenida del Conocimiento s/n, 18016 Granada, Spain; mcgamez@ujaen.es or or; 3 Department of Pharmacognosy, Faculty of Pharmacy, Cairo University, Kasr El-Aini Street, Cairo 11562, Egypt; essam.abdelsattar@pharma.cu.edu.eg; 4 Department of Analytical Chemistry, Faculty of Sciences, University of Granada, Avenida Fuentenueva s/n, 18071 Granada, Spain

**Keywords:** *Sesamum indicum* L., sesame cake, RP-HPLC–DAD–QTOF-MS, phenolic acids, lignans, flavonoids, agri-food residues

## Abstract

Agri-food residues represent a rich source of nutrients and bioactive secondary metabolites, including phenolic compounds. The effective utilization of these by-products in food supplements and the nutraceuticals industry could provide a way of valorization in the transition to becoming more sustainable. In this context, the present study describes the phenolic profiling of sesame (*Sesamum indicum* L.) cake using reversed-phase high-performance liquid chromatography coupled to diode array detection and quadrupole-time-of-flight-mass spectrometry. Compounds were characterized based on their retention time, UV spectra, accurate mass spectrometry (MS) and MS/MS data along with comparison with standards, whenever possible, and the relevant literature. The characterized compounds (112 metabolites) belong to several classes, namely, phenolic acids (hydroxybenzoic acids and hydroxycinnamic acids), flavonoids, and lignans. Moreover, organic acids and some nitrogenous compounds were characterized. The total phenol content and the antioxidant activity of the cake extract were determined. This study provides useful information for the valorization of by-products from the sesame oil industry.

## 1. Introduction

Pedaliaceae is considered a small family, with 14 genera and 70 species. It is natively distributed in the Old World and commonly known as the sesame family [1]. Sesame (*Sesamum indicum* L.) is a prominent oil crop that is cultivated all over the world. It is believed that sesame originates from India [2]. Nevertheless, it has been present in the Ancient Egyptian civilization since the third century BC, where the ancient Egyptian used it for soothing asthma [3].

Sesame seeds are considered a rich source of proteins, dietary fibers, carbohydrates, fats, and vitamins [2,4]. Several studies investigated the phytochemical composition of sesame seeds and/or oil with a focus on lignans. For instance, Dachtler and coworkers applied online liquid chromatography coupled to nuclear magnetic resonance and mass spectrometry (MS) to characterize lignans in sesame oil, whereas Grougnet and others isolated lignans from sesame seeds and perisperms, respectively [5,6]. Regarding other phytochemicals, Hassan studied the physical characteristics of two Egyptian cultivars of sesame as well as their content of phenolic acids while taking into account the effect of roasting of seeds. Botelho et al. applied supercritical fluid extraction to black sesame seeds. The extracts showed significant amounts of unsaturated fatty acids and phytosterols, which were subjected for neuroprotective studies [7]. Moreover, sesame seeds showed several biological activities *viz.* antioxidant, hypolipidemic, hypocholesterolemic, antidiabetic, anticancer, antihypertensive, and cardioprotective activities [2,8,9]. Thus, more studies are required to establish the chemical composition of sesame since, as far as we know, most of the published studies on sesame focused on a short list of compounds.

Today, new efforts are addressed to valorize agri-industrial by-products in different ways. This includes obtaining extracts rich in functional secondary metabolites, which can be useful in the sustainable development of functional food supplements and nutraceuticals [10]. In accordance with the Food and Agriculture Organization (FAO) of the United Nations statistics, the global production of sesame is higher than 7 million tons, and around 2 million tons of sesame oil are produced. Consequently, there is a growing interest in the utilization of the cake by-product, which represents nearly 70% of the total production of the seeds [11]. The cake could maintain a large part of the phytochemical composition of the seeds and, thus, their functionality.

Therefore, the objective of this study was to perform an untargeted metabolic profiling of sesame cake, mainly focused on the structural elucidation of phenolic compounds, through reversed-phase (RP) high-performance liquid chromatography (HPLC) coupled to diode array detection (DAD) and quadrupole-time-of-flight (QTOF)-MS. In addition, the antioxidant activity *via* measurement of the total phenol content (TPC) and the Trolox equivalent antioxidant capacity (TEAC) was assayed. These results give new insights into the phenolic composition of this undervalued sesame by-product.

## 2. Materials and Methods

### 2.1. Chemicals and Reagents

Methanol, *n*-hexane, acetone, acetonitrile, and glacial acetic acid were purchased from Fisher Chemicals (Thermo Fisher Scientific, Waltham, MA, USA). The solvents used for extraction and characterization were of analytical and MS grade, respectively. Ultrapure water was obtained with a Milli-Q system (Millipore, Bedford, MA, USA). Folin & Ciocalteu’s phenol reagent, sodium carbonate, ABTS (2,2′-azinobis (3-ethylbenzothiazoline-6-sulfonate)), Trolox (6-hydroxy-2,5,7,8-tetramethylchroman-2-carboxylic acid), potassium persulfate, l-tyrosine, and phenolic standards were purchased from Sigma-Aldrich (St. Louis, MO, USA), while l-tryptophan and l-phenylalanine were from Acros Organics (Morris Plains, NJ, USA). Kaempferide was purchased from Extrasynthese (Genay, France). The degree of purity of the standards was around 95% (*w*/*w*).

### 2.2. Samples Procurement and Extraction Procedures

The Egyptian sesame cultivar ‘Giza 32’ was kindly provided and identified by Agriculture Engineer Nadia Abdel-Azim, Egyptian Ministry of Agriculture and Land Reclamation (Giza, Egypt). Prior to the extraction, the seeds were ground (particle size around 1 mm) with Ultra Centrifugal Mill ZM 200, Retsch (Haan, Germany).

The first step of the extraction method was performed according to previous studies [12,13] with some modifications. For each one, 1 g of sample was firstly homogenized with 10 mL *n*-hexane and subjected to magnetic stirrer Agimatic-N (Jp Selecta, Barcelona, Spain) at room temperature for 30 min for defatting. Then defatted sesame cake was homogenized with 25 mL methanol/water (50:50, *v*/*v*) using an Ultra-TurraxIka T18 basic (Ika-Werke GmbH & Co. KG, Staufen, Germany), sonicated for 10 min at room temperature in an ultrasonic bath B3510 (40 kHz) (Branson, Danbury, CT, USA), and homogenized in the aforementioned magnetic stirrer at room temperature for 60 min. The mixture was finally centrifuged at 7155× *g* (8000 rpm) for 15 min and 5 °C using Sorvall ST 16 (Thermo Sci., ThermoFisher, Waltham, MA, USA) and the supernatant was collected. The pellet was re-extracted with 25 mL acetone/water (70:30, *v*/*v*). Both supernatants were combined and evaporated under vacuum using a rotary evaporator at 38 °C (Rotavapor R-200, BüchiLabortechnik, AG, Switzerland). Three independent extractions were performed. Finally, the dry cake extracts were dissolved in methanol/water (80:20, *v*/*v*), filtered (0.45 µm syringe filter, regenerated cellulose) and stored at −20 °C until analysis.

### 2.3. Analysis by RP-HPLC–DAD–ESI–QTOF-MS and -MS/MS

Analyses were made with an Agilent 1200 series rapid resolution (Santa Clara, CA, USA) equipped with a binary pump, an autosampler, and a diode array detector (DAD). Separation was carried out with a core-shell Halo C18 analytical column (150 mm × 4.6 mm, 2.7 μm particle size). The system was coupled to a 6540 Agilent Ultra-High-Definition (UHD) Accurate-Mass Q-TOF LC/MS equipped with an Agilent Dual Jet Stream electrospray ionization (Dual AJS ESI) interface.

Gradient elution was conducted with two mobile phases consisting of acidified water (0.5% acetic acid, *v*/*v*) (phase A) and acetonitrile (phase B) with a constant flow rate of 0.5 mL/min according to [13]. The gradient program was 0 min, 99% A and 1% B; 5.50 min, 93% A and 7% B; 11 min, 86% A and 14% B; 17.5 min, 76% A and 24% B; 22.50 min, 60% A and 40% B; 27.50 min, 0% A and 100% B; 28.5 min 0% A and 100% B; 29.5 min, initial conditions, which were finally maintained for 5.50 min for column equilibration (total run 35 min). The injection volume was 15 µL and three analyses were performed.

The operating conditions briefly were drying nitrogen gas temperature, 325 °C with a flow of 10 L/min; nebulizer pressure, 20 psig; sheath gas temperature, 400 °C with a flow of 12 L/min; capillary voltage, 4000 V; nozzle voltage, 500 V; fragmentor voltage, 130 V; skimmer voltage, 45 V; octapole radiofrequency voltage, 750 V. Data acquisition (2.5 Hz) in profile mode was governed *via* MassHunter Workstation software (Agilent technologies). The spectra were acquired in negative-ion mode over a mass-to-charge (*m/z*) range from 70 to 1500. The detection window was set to 100 ppm. Reference mass correction on each sample was performed with a continuous infusion of Agilent TOF biopolymer analysis mixture containing trifluoroacetic acid ammonium salt (*m/z* 112.9856 corresponding to the trifluoroacetic acid ion) and hexakis (1H, 1H, 3H-tetrafluoropropoxy) phosphazine (*m/z* 1033.9881 corresponding to the trifluoroacetic acid ammonium salt adduct ion). MS/MS experiments were performed in automatic mode, using the following collision energy values: *m/z* 100, 40 eV; *m/z* 500, 45 eV; *m/z* 1000, 50 eV; and *m/z* 1500, 55 eV.

MassHunter Qualitative Analysis B.06.00 (Agilent technologies) was used for data analysis following the strategy proposed by [10,12,14]. The characterization of compounds was performed by generation of candidate formula with a mass accuracy limit of 5 ppm. The MS score related to the contribution to mass accuracy, isotope abundance and isotope spacing for the generated molecular formula was set at ≥90. After the generation of the molecular formula, retention time (Rt), UV, and MS/MS spectra were also considered and compared with literature. Consequently, the following chemical structure databases were consulted: ChemSpider (http://www.chemspider.com), SciFinder Scholar (https://scifinder.cas.org), Reaxys (http://www.reaxys.com), PubChem (http://pubchem.ncbi.nlm.nih.gov), KNApSAcK Core System database (http://www.knapsackfamily.com/knapsack_jsp/top.html), METLIN Metabolite Database (http://metlin.scripps.edu), Phenol-Explorer (www.phenol-explorer.eu), Dictionary of Natural Products (http://dnp.chemnetbase.com), Phytochemical dictionary of natural products database [15] and tracing available literature *via* Egyptian Knowledge Bank (https://www.ekb.eg/). Confirmation was made through a comparison with standards, whenever these were available in-house. Moreover, the peak area obtained by MS for each compound was determined to estimate the abundance of each characterized phenolic compound.

### 2.4. Total Phenol Content (TPC) Assay

The TPC of the extracts was determined in triplicate colorimetrically by Folin–Ciocalteu reagent [16] and modified according to [17] in 96-well polystyrene microplates (Thermo Fisher) and using a Synergy MxMonochromator-Based Multi-Mode Microplate reader (Bio-Tek Instruments Inc., Winooski, VT, USA). The absorbance of the solution was measured at a wavelength of 760 nm after incubation for 2 h in the dark and compared with a calibration curve of serially diluted gallic acid (GA). The results were expressed as GA equivalents (GAE). Analyses were done in triplicate.

### 2.5. Trolox Equivalent Antioxidant Capacity Assay

TEAC absorbance measurements were performed using the aforementioned microplate reader and following the procedure described by [12]. Briefly, ABTS^+^ was produced by reacting ABTS stock solution with 2.45 mM potassium persulfate (final concentration). The mixture was kept in dark at room temperature for 24 h and the solution diluted with water until reaching an absorbance value of 0.70 (±0.03) at 734 nm. Afterwards, 300 μL of this solution and 30 μL of the cake extract were mixed and measured. Absorbance reading was compared to a standard calibration curve of Trolox and the results expressed as equivalents of Trolox (TE). Caffeic acid was used as a positive control. Analyses were done in triplicate.

## 3. Results and Discussion

### 3.1. Metabolic Profiling of Sesame Cake by RP-HPLC–DAD–QTOF-MS and -MS/MS

**Figure 1:**
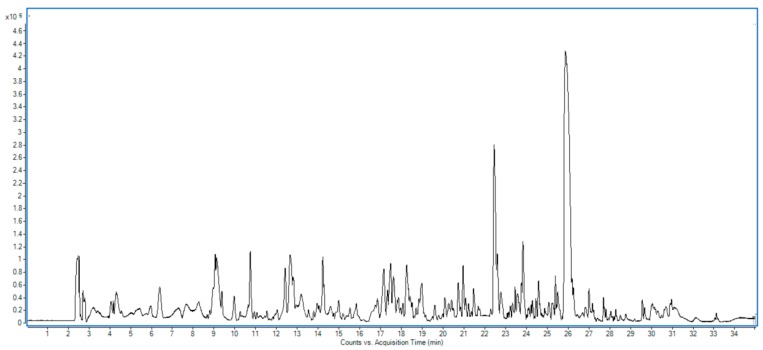
Base peak chromatogram of the cake of the Egyptian cultivar of sesame ‘Giza 32’.

The sesame cake was subjected to core–shell RP-HPLC–DAD–ESI–QTOF-MS and -MS/MS analysis in negative ionization mode, providing Rt, experimental *m/z*, generated molecular formulae, mass error in ppm, mass score, double bond equivalents (DBE), UV maxima, and tandem mass fragments. For clarification, Table 1 and Table 2 illustrate the aforementioned parameters for phenolic compounds and non-phenolic compounds, respectively. Moreover, Tables S1 and S2 (supplementary material) also detail metabolites class/subclass, plant species, family, and previously reported literature for phenolic compounds and non-phenolic compounds, respectively. The total number of characterized metabolites was 112, including 92 metabolites that were reported for the first time in sesame, with 20 new proposed structures. Figure 1 shows the base peak chromatogram of the cake extract, showing its complexity.

#### 3.1.1. Hydroxybenzoic Acids

Qualitatively, phenolic acids were the most abundant phenolic compounds by 40 metabolites. They are divided into hydroxybenzoic acids (13) and hydroxycinnamic acids (27).

Concerning hydroxybenzoic acids, they can be classified into non-hydroxylated (benzoic acid), mono-hydroxylated (*p*-hydroxybenzoic acid), di-hydroxylated (gentisic, protocatechuic, and vanillic acid derivatives), and tri-hydroxylated (gallic acid and syringic acid derivatives). It bears noting that five of them were confirmed with standards. In brief, *m/z* 121.03 exerted a neutral loss of CO and UV absorbance at λ_max_ 272 nm. It was characterized as benzoic acid and has been previously reported in sesame [4]. The compound with a *m/z* value of 137.02 was confirmed to be *p*-hydroxybenzoic acid upon comparison with a standard. Similarly, two compounds showed a molecular formula of C_7_H_6_O_4_ and *m/z* of 153.02. By comparing with standards, they were confirmed as protocatechuic acid (3,4-dihydroxybenzoic acid) and gentisic acid (2,5-dihydroxybenzoic acid).

**Figure 2:**
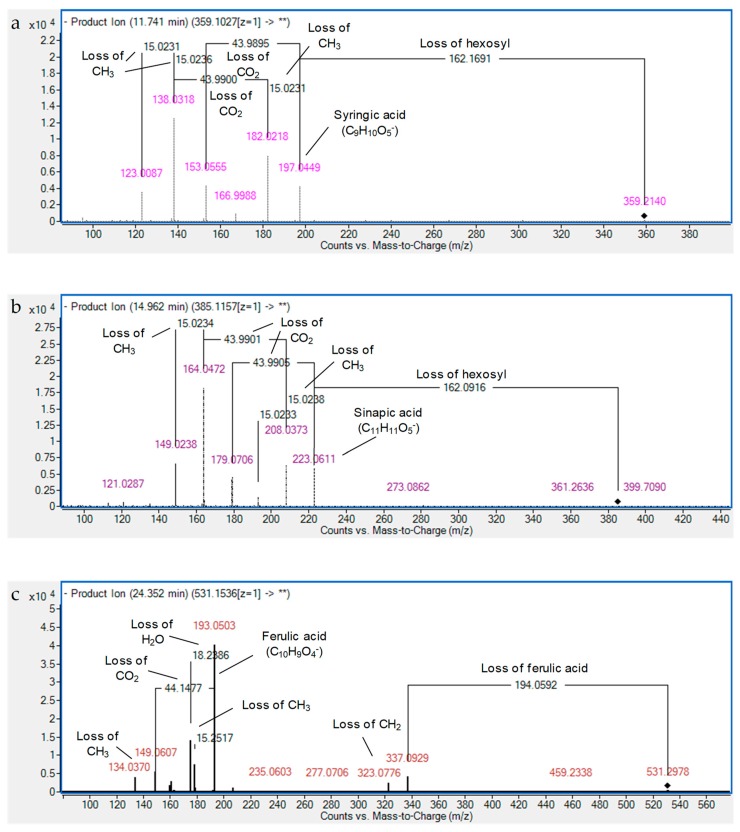
Fragmentation patterns of (**a**) syringic acid hexoside, (**b**) sinapic acid hexoside, and (**c**) diferuloyl hexoside.

In regard to *O*-methylated derivatives of dihydroxybenzoic acids, five derivatives of vanillic acid were characterized. Vanillic acid was observed with *m/z* 167.04, and was confirmed with standard. It has been reported before in sesame [18]. In addition, three isomers of vanillic acid hexoside (C_14_H_18_O_9_) were observed at Rt 9.02, 10.28, and 10.66 min. They revealed the neutral loss of the hexose moiety (162 Da) and CO_2_ (44 Da), complying with the typical decarboxylation of phenolic acids [10,12,17]. They were reported for the first time in *S. indicum*, nevertheless, they were reported in the genus *Sesamum* in accordance with the Dictionary of Natural Products database (Table S1). Vanillic acid pentoside hexoside was characterized with *m/z* 461.13 and sequential loss of pentose (*m/z* 329.09), hexose (*m/z* 167.03, i.e., vanillic acid ion), and methyl (*m/z* 153.02) moieties. It was detected for the first time in sesame (Table 1 and Table S1). Concerning tri-hydroxylated benzoic acids, both gallic and syringic acids were observed with *m/z* 169.01 and 197.05, respectively. These assignments were confirmed with standards. It is worth noting that the compound with an *m/z* value of 329.09 and molecular formula C_14_H_18_O_9_ exerted the neutral loss of pentose with an aglycone fragment of (*m/z* 197.05) followed by fragmentation of syringic acid. It was tentatively identified as syringic acid pentoside and proposed as a new structure. Similarly, compound at *m/z* 359.10 (C_15_H_20_O_10_) showed a neutral loss of a hexose moiety with syringic acid fragmentation pattern. In this way, two consecutive losses of CH_3_ were observed until fragment 166.10, indicating methoxy substituents. Moreover, the loss of CO_2_ from the carboxyl moiety was also observed (*m/z* 153.06 and 138.03) followed by losses of CH_3_ (Figure 2a) [17]. Therefore, it was identified as syringic acid hexoside, which was described for the first time in sesame.

#### 3.1.2. Hydroxycinnamic Acids

Regarding hydroxycinnamic acid, it was found in free form, conjugated with quinic acid or with sugars. The occurrence of *p*-coumaric acid, *m*-coumaric acid, chlorogenic acid (caffeoylquinic acid), caffeic acid, ferulic acid, and sinapic acid were unequivocally confirmed with standards enabling characterization validation, which was in agreement with previous studies [19]. It is worth noting that both *m*-coumaric acid and chlorogenic acid were described for the first time in sesame. Moreover, cinnamic acid (21, *m/z* 147.05) was observed with the neutral loss of CO_2_ from the carboxylate moiety and water (Table 1 and Table S1).

Five isomers of *p*-coumaric acid hexosides were detected expressing the neutral loss of hexose moieties releasing aglycones of *m/z* 163.04 followed by decarboxylation (*m/z* 119.05), except for the hydrated form (M−H+H_2_O) and, hence, dehydration occurred firstly [13]. In the same manner, three isomers of ferulic acid hexoside (C_16_H_20_O_9_, *m/z* 355.10) and sinapic acid hexoside (C_17_H_22_O_10_, *m/z* 385.11) were observed (Table 1 and Table S1). As an example, the fragmentation of the latter compound is shown in Figure 2b. Another compound was described as ferulic acid dihexoside (sibiricose A5). In addition, seven novel hydrocinnamic acids were tentatively identified as ferulic acid pentoside isomers (I–III), sinapic acid deoxyhexoside hexoside isomers (I–III), and diferuloyl hexoside (Table 1 and Table S1). The latter was characterized by the presence of fragment ions at *m/z* 337.0929 (C_16_H_17_O_8_^−^) and 193.0503 (C_10_H_9_O_4_^−^), i.e., feruloylhexosyl and ferulic acid, respectively. Ferulic acid ion showed the typical decarboxylation and demethylation of this type of compound in MS/MS (Figure 2c), as for sinapic acid. Remarkably, most of these compounds are described for the first time in sesame (Table S1).

Moreover, caffeoyl derivatives were found. Based on molecular formula (C_21_H_28_O_13_) and fragmentation pattern with successive loss of deoxyhexose and hexose, the caffeic acid derivate cistanoside F (C_21_H_28_O_13_) was characterized. Furthermore, three caffeoylphenylethanoid derivatives were observed. Verbascoside and isoverbascoside expressed the neutral loss of caffeoyl moieties and deoxyhexoses through the presence of the ion *m/z* 461.17 and *m/z* 315.11, respectively. β-Hydroxyverbascoside was identified with similar fragmentation pattern as of verbascoside with additional loss of hydroxyl group (16 Da) [19]. These caffeoyl derivatives were isolated before from sesame [20,21].

#### 3.1.3. Lignans

Lignans are natural phenolic compounds that possess several biological activities, especially antioxidant and estrogenic activities. A simple lignan is composed of two phenyl propanoid derivatives (C6–C3) linked through a *β*–*β*-linkage [22]. In this context, 18 lignan derivatives were identified in cake of sesame, which belonged to the furofuran subclass of lignans. As a matter of fact, the in-depth analysis of tandem MS data made it possible to preliminary predict lignan structures, whose presence in sesame were not discovered and could yet contribute to its bioactivity (Table 1). For that, studies using tandem MS/MS were consulted for analog structures [22,23,24,25]. All of them were glycosides, and the loss of each sugar was observed until the product ions of the aglycones were released and hence observed [22].

**Figure 3:**
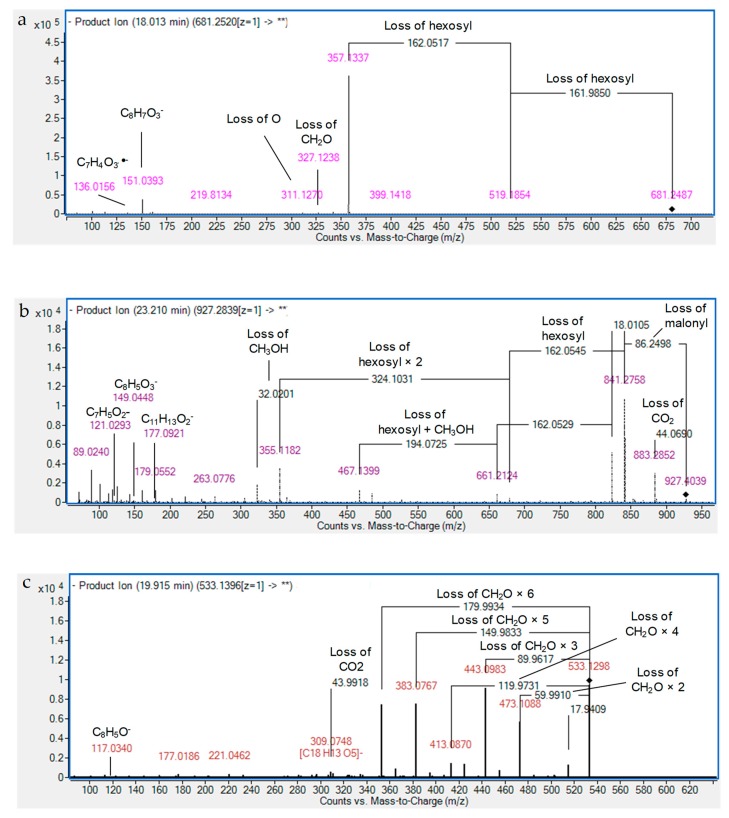
Patterns of (**a**) pinoresinol dihexoside I, (**b**) xanthoxylol malonyl trihexoside, and (**c**) apigenin di-*C*-pentoside II.

Briefly, three isomers of pinoresinol dihexoside were detected showing subsequent losses of two hexosyl moieties with aglycone fragmentation showing the fragment *m/z* 151.04 (C_8_H_7_O_3_^−^) due to cleavage of the tetrahydrofuran ring followed by methyl loss from the guaiacyl moiety [22,24,25] (Table 1 and Table S1, Figure 3a). Similarly, two isomers, (*m/z* 767.24, C_35_H_44_O_19_), resembled the aforementioned fragmentation with additional loss of a malonyl moiety (CO_2_ and an acetyl moiety CH_2_CO) (86 Da). Consequently, they were characterized as pinoresinol malonyl dihexoside (I–II). In the same manner, xanthoxylol trihexoside and xanthoxylol malonyl trihexoside were tentatively characterized with the observation of the loss of CH_3_OH (32 Da) (*m/z* 323.10). In addition, minor fragments were observed at *m/z* 149.05 from the aglycone corresponding to methylenedioxyphenyl-CO (C_8_H_5_O_3_^−^) [23] and the counterpart after the loss of CO (*m/z* 177.09). The ion *m/z* 121.03 (C_7_H_5_O_2_^−^, methylenedioxyphenyl) was also observed according to [22,23], as well more abundant ions from sugars such as *m/z* 179.06 (hexose, C_6_H_12_O_6_), 161.05 (hexosyl, C_6_H_10_O_5_), and 89.02 (C_3_H_5_O_3_^−^). For clarification, Figure 3b illustrates the fragmentation pattern of xanthoxylol malonyl trihexoside. Although these glycosides have been reported here for the first time, and aglycone was characterized by Fukuda et al. [25].

In regard to sesaminol derivatives, two isomers of sesaminol trihexoside (I–II) and sesaminol tetrahexoside (I–II) were identified with the observation of *m/z* 149.05 characterizing furofurano lignans [23], as commented upon before, as well as the counterpart at *m/z* 219.06 (C_12_H_11_O_4_^−^). Moreover, two isomers of sesaminol dipentoside were identified, also showing a fragment at *m/z* 135.03; i.e.,methylenedioxyphenyl–CH_2_ (C_8_H_7_O_2_^−^) [23]. The acetic acid adduct of sesamolinol hexoside (*m/z* 593.19, C_28_H_34_O_14_) and three new isomers of sesamolinol dipentoside were observed with the observation of the aglycone at *m/z* 371.11 (Table 1 and Table S1). In the first case, fragment ions at *m/z* 138.0323 (C_7_H_6_O_3_^−^) and *m/z* 233.0817 (C_13_H_13_O_4_^−^) were detected, corresponding to the fragmentation of the aglycone structure, while in the rest of cases, the fragmentation of the aglycone was poor under the MS/MS conditions used for the assay. Finally, hydroxysesamolin trihexoside was tentatively identified with the presence of aglycone at *m/z* 385.09. The aglycone part showed MS/MS product ions at 137.0244 (C_7_H_5_O_3_^−^), which could indicate a similarity to sesamolinol structure, but also product ions at 165.0192 (C_8_H_5_O_4_^−^) and 149.0452 (C_8_H_5_O_3_^−^), which could indicate the presence of a hydroxylated methylenedioxyphenyl–CO moiety.

#### 3.1.4. Coumarins

Regarding coumarins, umbelliferone (7-hydroxycoumarin) was unequivocally confirmed with a standard.

#### 3.1.5. Flavonoids

A total of 26 flavonoids were characterized in the sesame cake extract, being classified mainly into a flavan-3-ol, flavanones (2), flavones (15), and flavonols (8) (Table 1). It is worth noting that (−)-epicatechin, naringenin, luteolin 7-*O*-β-d-glucopyranoside, luteolin, apigenin, quercetin, rutin, quercetin 3-*O*-β-d-glucopyranoside, quercetin 3-*O*-β-d-galactopyranoside, quercetin 3-*O*-rhamnopyranoside, myricetin, kaempferol, and kaempferide were identified through comparison with standards. All of them were described for the first time in the genus *Sesamum*, except for apigenin and luteolin 7-*O*-β-d-glucopyranoside, according to the phytochemical dictionary database [15].

Additionally, the fragmentation pattern of compound at *m/z* 255.07 (Rt 29.81 min, C_15_H_12_O_4_) revealed the common fragment ion released after retro Diels–Alder fission and retrocyclization at *m/z* 151.00 (C_7_H_3_O_4_) (^1,3^A^−^) by this type of compound [26]. Moreover, the ion fragment with *m/z* at 103.05 (C_8_H_7_^−^) could be derived from band B (^1,3^B^−^). Besides the fragmentation pattern, it showed UV absorbance at λ_max_ 288 nm, suggesting a flavanone nucleus [12]. Therefore, it was tentatively identified as pinocembrin, which was described for the first time in sesame.

The occurrence of *C*-glycosides of flavones was noticed with 12 derivatives of either luteolin or apigenin, which were observed for the first time in sesame. They were characterized by the presence of prominent fragment ions after the characteristic sequential loss of 90 (C_3_H_6_O_3_) and/or 120 Da (C_4_H_8_O_4_), in agreement with previous studies [12,14,17,19,27]. As an example, two isomers of luteolin *C*-hexoside were identified, exerting characteristic fragments at *m/z* 357.06 and 327.05, respectively. Similarly, three isomers of luteolin *C*-deoxyhexoside-*C*-hexoside were tentatively identified based on comparing their fragmentation pattern and UV absorbance with reported literature [27]. As for apigenin derivative, five isomers of apigenin *C*-pentoside-*C*-hexoside were observed, showing fragmentation patterns and UV absorbance of *C*-flavones as described in reported studies [15,17]. Moreover, a minor fragment ion at *m/z* 117.03 (C_8_H_5_O) (^1,3^B^−^) was observed, suggesting that the aglycone is apigenin. Similarly, two isomers of apigenin di-*C-*pentoside were tentatively characterized. As Figure 3c shows, the consequent neutral loss of sugar fragments (30–180 Da) was observed (Table 1 and Table S1, Figure 3c).

#### 3.1.6. Others (Non-Phenolic Compounds)

A total of 17 organic acids were observed in the cake of the sesame, namely gluconic/galactonic acids, citric acid (I–III), malic acid (I–II), citramalic acid, itaconic acid, (−)-3-dehydroshikimic acid, quinic acid (I–II), pantothenic acid (I–II), isopropylmalic acid (I–II), and azelaic acid. Their fragmentation patterns were in agreement with reported studies [12,14,17,19,28,29,30] (Table 2 and Table S2). All of the identified organic acids are reported for the first time in sesame.

Regarding nitrogenous compounds, it is worth mentioning that five amino acids were characterized, *viz.* asparagine, leucine/isoleucine, tyrosine, and phenylalanine. Their fragmentation patterns were characterized by deamination and/or decarboxylation [13,15,17]. In addition, both tyrosine and phenylalanine were confirmed with standards. Furthermore, a peptide was observed (Rt 6.29 min, *m/z* 611.1454, C_20_H_32_N_6_O_12_S_2_) exerting the loss of a glutathione moiety (*m/z* 306.08) followed by a loss of SH_2_ from the cysteinyl group. Finally, the product ion of the glutamyl moiety was observed at *m/z* 128.04. It was compared with data on the METLIN database to be described as oxidized glutathione (GSSG), indicating the presence of reduced glutathione (GSH) in the cake of sesame, which is easily auto-oxidized to GSSG during sample preparation and/or analysis [31]. In fact, GSH is considered to be a powerful cellular antioxidant that prevents oxidative stress in biological systems and, hence, prevents the onset and progression of many serious diseases such as diabetes mellitus, cancer, and Alzheimer’s disease [31]. Furthermore, a derivative of tryptophan (*m/z* 529.18, C_26_H_30_N_2_O_10_) was observed as well as succinyladenosine (*m/z* 382.10, C_14_H_17_N_5_O_8_), a nucleoside derivative. It bears noting that the characteristic tetrasaccharide sesamose could be the ion with *m/z* 665.21, presenting subsequent losses of hexosyl moieties in MS/MS (Table 2 and Table S2).

### 3.2. TPC, TEAC, and Phenolic Abundance

The extract of sesame cake showed a total phenol content of 1.9 ± 0.3 mg GAE/g cake extract. In fact, this value is even beyond results by Mohadaly et al. [18], where total phenol contents were assayed of single different solvents cake extracts of the Egyptian cultivar ‘Shandweel-3’. The value of TPC ranged from 0.1 (petroleum ether extract) to 0.8 (methanol extract) mg GAE/g cake extract. This could be attributed to the combined solvent extraction accompanied with ultra-sonication, which enhances the extraction process [12,32]. In regard to the TEAC assay, the extract expressed a value of 2.65 ± 0.08 µmol TE/g of cake extract. In fact, Janu et al. [33] focused on the antioxidant activity of the sesame oil, which was found to be 0.004 µg TE/mL oil (i.e., around 0.02 µmol TE/g oil) indicating the value of the cake as an agri-industrial by-product that needs further attentions for its antioxidant potential as well as other biological activities.

**Figure 4:**
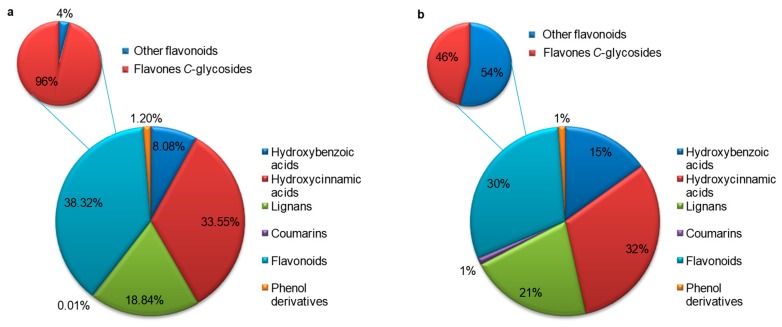
Summary of the characterization results on sesame phenolic compounds: (**a**) relative abundance (%) and (**b**) qualitative classification (%).

To evaluate the contribution of phenolic compounds, a summary of the characterization results is shown in Figure 4. In the perspective of subclasses, flavonoids were the most abundant, representing 38.3% of the total characterized phenolic metabolites followed by hydroxycinnamic acids and then lignans (Figure 4a). Similarly, flavonoids and hydroxycinnamic acids were also the most representative families in qualitative terms (Figure 4b).

The exploration of alternative strategies for the treatment of many chronic diseases such as cancer, diabetes, and heart and liver diseases continues to attract scientists in discovering drugs derived from plant origins [34,35,36]. In fact, there is growing attention in the valorization of agri-food residues to provide new functional ingredients with bioactivities for sustainability of the agri-industry. It bears noting that such by-products represent around 40% of total plant foods [10,37]. For that, the elucidation of the potential bioactive phytochemicals is a requirement. In this regard, the application of UHPLC–QTOF-MS enabled us to characterize 86 phenolic compounds in sesame cake and, hence, as far as we know, this is the first study providing comprehensive phenolic profiling of sesame cake. In addition, the antioxidant activity of the sesame cake extract was determined by the TEAC method. In this regard, furofurano lignans possess anticancer, cardiovasculoprotective, neuroprotective, antioxidant, and anti-inflammatory activities. Moreover, they are metabolized by gut microflora into enterolactone and enterodiol, which are considered phytoestrogens [38]. Thus, these compounds could contribute to the antioxidant activity of the extract.

In this line, a previous study on sesame cake showed that the main contributors to the antioxidant activity were sesamol and water- (sesaminol tri- and di-glucoside) and lipid-soluble lignans (sesamin and sesamolin), but the extraction procedure was based on Soxhlet extraction with methanol [39]. In this sense, our results revealed the presence of sesamol and a wider range of lignan glycosides, but there were no free lignans, which could be due to the use of more polar extraction conditions and the removal of the fatty phase. Moreover, *C*-glycosides were the most abundant compounds both as a subclass, accounting for around 37.3% in relative abundance, and individually, i.e., apigenin *C*-pentoside-*C*-hexoside I (8.8%) followed by luteolin *C*-deoxyhexoside-*C*-hexoside III (7.0%), luteolin *C*-deoxyhexoside-*C*-hexoside II (6.7%), and apigenin *C*-pentoside-*C*-hexoside IV (5.1%). As a matter of fact, it seems that *C*-glycosylation enhances antioxidant capacity, where the hydroxyl group and metal chelation sites of flavones are free [14,40]. Thus, these compounds could be the highest contributors to the antioxidant activity of the extract, agreeing with Zhou et al. [41]. These authors highlighted that the antioxidant activity of sesame cake extracts was associated with the total content of flavonoids, but these compounds were not characterized. Furthermore, oxidized glutathione was detected for the first time in the cake of sesame, which could be produced from reduced glutathione during sample preparations. It is considered a strong marker for the antioxidant potential of this agri-industrial byproduct.

## 4. Conclusions

In this study, core–shell RP-HPLC–DAD–ESI–QTOF-MS and -MS/MS methods were employed to analyze the cake of the Egyptian cultivar of sesame ‘Giza 32’. A total of 112 metabolites were characterized in sesame cake, and among them, 86 were phenolic compounds. The observed lignans were of furofurano type and, among them, 12 lignans are considered to be new proposed structures. Moreover, this is the first report showing the conjugation of malonyl moieties to lignans in Pedaliaceae. With regard to the characterized flavonoids, they were classified into flavones (15), flavonols (8), flavanones (2), and a flavan-3-ol. *C*-Glycosides of flavones have been reported here for the first time in sesame. This type of flavonoid was the most abundant. Furthermore, the antioxidant activity of the sesame cake extract was determined by TEAC method and, hence, our results suggest that not only sesamol and lignans, but also *C*-glycosides and other compounds could contribute to this bioactivity. Consequently, further studies are required for the development of food supplements and nutraceuticals from sesame cake to widen its applicability and to move into a more sustainable industry with zero waste.

## Figures and Tables

**Table 1:** Phenolic compounds characterized in the cake of the Egyptian cultivar of *Sesamum indicum* L. ‘Giza 32’.

Number	Rt (min)	Experimental *m/z* ^a^ [M − H]^−^	Theoretical Mass (M)	Molecular Formula	Error (ppm)	Error (mDa)	Score	Main Fragments	DBE	UV (nm)	Proposed Compound ^c^
1	7.33	169.0140	170.0215	C_7_H_6_O_5_	2.09	0.36	84.05	N.D.	5	N.D.	Gallic acid *
2	8.74	137.0242	138.0316	C_7_H_6_O_3_	0.51	0.07	96.91	N.D.	5	N.D.	Sesamol
3	9.02	329.0881	330.0951	C_14_H_18_O_9_	−0.5	−0.2	92.03	167.0351, 123.0456	6	256sh, 286	Vanillic acid hexoside I
4	9.10	147.0454	148.0524	C_9_H_8_O_2_	−1.2	−0.3	98.8	103.0556, 85.0290	6	255	Cinnamic acid
5	9.56	329.0878	330.0951	C_14_H_18_O_9_	0.0	0.0	96.43	197.0457, 153.0193	6	N.D.	**Syringic acid pentoside**
6	9.91	343.1036	344.1107	C_15_H_20_O_9_	−0.2	−0.1	99.74	163.0401, 119.0498	6	N.D.	***p*-Coumaric acid hexoside I (in hydrated form)**
7	10.28	329.0879	330.0951	C_14_H_18_O_9_	−1.0	−0.3	94.93	167.0349, 152.0117, 123.0450, 108.0218	6	257sh, 285	Vanillic acid hexoside II
8	10.66	329.0882	330.0951	C_14_H_18_O_9_	−1.0	−0.3	97.69	167.0349, 152.0113, 123.0451, 108.0217	6	255sh, 285	Vanillic acid hexoside III
9	11.20	461.1302	462.1373	C_19_H_26_O_13_	0.0	0.0	94.27	329.0876, 299.0775, 167.0345, 152.0194	7	N.D.	Vanillic acid pentosidehexoside
10	11.33	153.0194	154.0266	C_7_H_6_O_4_	−0.64	−0.1	84.11	N.D.	5	257, 283sh	3,4-Dihydroxybenzoic acid * (protocatechuic acid)
11	11.83	359.0982	360.1057	C_15_H_20_O_10_	0.4	0.2	99.54	197.0449, 182.0218, 166.9988, 153.0555, 138.0318, 123.0087	6	267	Syringic acid hexoside
12	12.35	325.0932	326.1002	C_15_H_18_O_8_	−0.6	−0.2	99.12	163.0399, 119.0500	7	287	*p*-Coumaric acid hexoside II
13	12.72	325.0932	326.1002	C_15_H_18_O_8_	−0.6	−0.2	99.12	163.0419, 119.0503	7	286	*p*-Coumaric acid hexoside III
14	13.58	487.1456	488.1530	C_21_H_28_O_13_	−0.08	−0.04	80.23	341.1085, 179.0544	8	270	Caffeic acid hexosidedeoxyhexoside (cistanoside F)
15	13.93	355.1039	356.1107	C_16_H_20_O_9_	−1.0	−0.4	99.18	193.0504, 178.0268, 134.0378, 119.0489	7	228, 288, 310	Ferulic acid hexoside isomer I
16	14.22	355.1038	356.1107	C_16_H_20_O_9_	−0.7	−0.3	98.93	193.0507, 178.0269, 134.0374, 119.0513	7	228, 288, 310	Ferulic acid hexoside isomer II
17	14.30	153.0193	154.0266	C_7_H_6_O_4_	0.33	0.05	85.78	N.D.	5	N.D.	2,5-Dihydroxybenzoic acid * (gentisic acid)
18	14.89	137.0242	138.0317	C_7_H_6_O_3_	−0.1	0.0	93.21	N.D.	5	272	*p*-Hydroxybenzoic acid *
19	14.96	385.1136	386.1213	C_17_H_22_O_10_	−0.5	−0.2	87.56	223.0611, 208.0373, 179.0706, 164.0472, 149.0238	7	290	Sinapic acid hexoside
20	14.99	353.0878	354.0951	C_16_H_18_O_9_	0.7	0.3	99.38	N.D.	8	278	Caffeoylquinic acid * (chlorogenic acid)
21	15.12	531.1719	532.1792	C_23_H_32_O_14_	0.0	0.1	96.82	385.1140, 223.0610, 179.0706, 165.0551, 150.0315	8	283	**Sinapic acid deoxyhexosidehexoside I**
22	15.16	517.1560	518.1635	C_22_H_30_O_14_	0.1	0.0	95.85	193.0501, 175.0395, 149.0606, 134.0367	8	277	Ferulic acid dihexoside (sibiricose A5)
23	15.58	325.0930	326.1002	C_15_H_18_O_8_	−0.30	−0.10	97.64	163.0398, 121.0293, 119.0498	7	228, 276	*p*-Coumaric acid hexoside IV
24	15.74	325.0930	326.1002	C_15_H_18_O_8_	−0.38	−0.12	99.22	163.0391, 119.0498	7	282, 312	*p*-Coumaric acid hexoside V
25	16.61	325.0932	326.1002	C_15_H_18_O_8_	−0.5	−0.2	97.58	193.0508, 134.0375	7	291	**Ferulic acid pentoside I**
26	16.75	167.0356	168.0426	C_8_H_8_O_4_	−3.59	−0.60	94.34	N.D.	5	232, 264, 293	Vanillic acid *
27	16.81	355.1050	356.1107	C_16_H_20_O_9_	−3.8	−1.4	93.17	193.0506, 178.0274, 134.0378, 119.0502	7	228, 288	Ferulic acid hexoside isomer III
28	16.88	593.1516	594.1585	C_27_H_30_O_15_	−0.6	−0.4	98.31	503.1189, 473.1090, 383.0773, 353.0666	13	280, 323	Luteolin*C*-deoxyhexoside*C*-hexoside I
29	17.01	179.0349	180.0422	C_9_H_8_O_4_	0.6	0.1	86.26	N.D.	6	233, 273	Caffeic acid *
30	17.15	593.1518	594.1585	C_27_H_30_O_15_	−1.0	−0.6	98.52	533.1313, 503.1623, 473.1085, 383.0772, 353.0664	13	272, 330	Luteolin*C*-deoxyhexoside*C*-hexoside II
31	17.44	197.0453	198.0528	C_9_H_10_O_5_	1.3	0.3	80.31	182.0218, 166.9984, 153.0182, 123.0088	5	273	Syringic acid *
32	17.51	593.1522	594.1585	C_27_H_30_O_15_	−1.7	−1.0	97.71	533.1290, 503.1186, 473.1084, 383.0770, 353.0663	13	272, 330	Luteolin*C*-deoxyhexoside*C*-hexoside III
33	17.62	531.1724	532.1792	C_23_H_32_O_14_	−0.8	−0.4	98.13	208.0329, 165.0560, 150.0323	8	278	**Sinapic acid deoxyhexosidehexoside II**
34	17.73	289.0718	290.0790	C_15_H_14_O_6_	−0.33	−0.10	85.47	N.D.	9	234, 272	(-)-Epicatechin *
35	17.83	531.1733	532.1792	C_23_H_32_O_14_	−2.1	−1.1	96.91	165.0558, 150.0322	8	277	**Sinapic acid deoxyhexosidehexoside III**
36	17.96	681.2400	682.2473	C_32_H_42_O_16_	0.00	0.00	99.86	519.1854, 357.1337, 342.1101, 327.1238, 151.0393, 136.0156	12	280	Pinoresinol dihexoside I
37	18.24	563.1415	564.1479	C_26_H_28_O_14_	−1.65	−0.93	96.8	545.1296, 503.1192, 473.1084, 443.0983, 413.0873, 383.0771, 353.0666, 117.0336	13	272, 327	Apigenin *C*-pentoside-*C*-hexoside I
38	18.30	121.0296	122.0368	C_7_H_6_O_2_	−1.21	−0.15	99.65	92.0266	5.5	272	Benzoic acid
39	18.67	563.1413	564.1479	C_26_H_28_O_14_	−1.01	−0.57	98.77	545.1297, 503.1188, 473.1086, 443.0983, 413.0876, 383.0766, 353.0661, 117.0351	13	272, 326	Apigenin *C*-pentoside-*C*-hexoside II
40	18.83	563.1414	564.1479	C_26_H_28_O_14_	−1.19	−0.67	98.47	545.1292, 503.1186, 473.1081, 443.0976, 413.0874, 383.0766, 353.0661, 117.0337	13	269, 337	Apigenin *C*-pentoside-*C*-hexoside III
41	18.90	447.0938	448.1006	C_21_H_20_O_11_	−2.10	−0.94	96.95	357.0618, 327.0510, 297.0405, 285.0407	12	272, 332	Luteolin *C*-hexoside I
42	18.93	563.1412	564.1479	C_26_H_28_O_14_	−0.97	−0.55	98.82	545.1296, 503.1186, 473.1082, 443.0976, 413.0874, 383.0768, 353.0664, 117.0346	13	272, 333	Apigenin *C*-pentoside-*C*-hexoside IV
43	19.18	533.1303	534.1373	C_25_H_26_O_13_	−0.45	−0.24	98.84	N.D.	13	274, 320sh	Apigenin di-*C*-pentoside I
44	19.44	563.1411	564.1479	C_26_H_28_O_14_	−1.08	−0.61	98.75	545.1300, 503.1206, 473.1087, 443.0980, 413.0875, 383.0768, 353.0668	13	N.D.	Apigenin *C*-pentoside-*C*-hexoside V
45	19.48	325.0930	326.1002	C_15_H_18_O_8_	−0.1	0.0	99.16	193.0506, 175.0973	7	N.D.	**Ferulic acid pentoside II**
46	19.60	447.0945	448.1006	C_21_H_20_O_11_	−2.35	−1.05	96.73	357.0611, 327.0507, 297.0404, 285.0400	12	265, 334	Luteolin *C*-hexoside II
47	19.85	767.2402	768.2477	C_35_H_44_O_19_	0.07	0.06	91.82	723.2460, 357.1341, 342.1102, 327.1236, 151.0399, 136.0165	14	276	**Pinoresinol malonyl dihexoside I**
48	19.92	533.1304	534.1373	C_25_H_26_O_13_	−1.49	−0.80	97.89	515.1191, 503.1164, 473.1088, 443.0983, 413.0870, 383.0101, 353.0667, 117.0340	13	277	Apigenin di*-C*-pentoside II
49	19.95	767.2405	768.2477	C_35_H_44_O_19_	−0.20	−0.15	97.21	723.2656, 681.2187, 561.1957, 357.1346, 342.1104, 327.1236, 151.0399, 136.0165	14	279	**Pinoresinol malonyl dihexoside II**
50	20.33	163.0399	164.0473	C_9_H_8_O_3_	−0.14	−0.02	96.2	119.0501	6	284	*p*-Coumaric acid *
51	20.52	609.1463	610.1534	C_27_H_30_O_16_	−0.32	−0.19	96.93	301.0825, 178.9975, 151.0029	13	N.D.	Quercetin 3-*O*-rutinoside (rutin) *
52	21.06	681.2399	682.2473	C_32_H_42_O_16_	0.50	0.34	97.7	519.1519, 357.1325, 327.0853, 339.0883, 309.0763, 151.0387, 137.0249	12	280	Pinoresinol dihexoside II
53	21.13	463.0884	464.0955	C_21_H_20_O_12_	−0.54	−0.25	96.41	N.D.	12	N.D.	Quercetin 3-*O*-β-d-glucopyranoside *
54	21.23	623.1994	624.2054	C_29_H_36_O_15_	−1.64	−1.02	97.45	461.1689, 315.1084, 297.0981, 179.0351, 161.0240, 153.0567, 135.0452, 113.0244	12	280, 308	Verbascoside
55	21.27	639.1993	640.2003	C_29_H_36_O_16_	−0.03	−0.02	96.46	477.1427, 331.0809, 297.0764, 179.0339, 161.0453	12	282	β-Hydroxyverbascoside (campneoside II)
56	21.30	463.0884	464.0955	C_21_H_20_O_12_	−1.11	−0.52	97.47	N.D.	12	N.D.	Quercetin 3-*O*-β-D-galactopyranoside*
57	21.36	447.0929	448.1006	C_21_H_20_O_11_	0.77	0.34	81.14	N.D.	12	N.D.	Luteolin 7-*O*-β-D-glucopyranoside *
58	21.48	223.0611	224.0685	C_11_H_12_O_5_	0.90	0.20	83.94	N.D.	6	N.D.	Sinapic acid *
59	21.54	193.0506	194.0579	C_10_H_10_O_4_	−0.4	−0.1	98.4	178.0271, 134.0371, 119.0502	7	230, 295sh, 322	Ferulic acid *
60	22.09	161.0245	162.0317	C_9_H_6_O_3_	−1.2	−0.2	84.29	N.D.	7	N.D.	7-Hydroxycoumarin * (umbelliferone)
61	22.29	1017.3112	1018.3165	C_44_H_58_O_27_	−1.44	−1.46	97.58	855.2379, 693.1894, 369.0920, 323.0935, 221.0642, 219.0625, 179.0543, 161.0422, 149.0425, 143.0341	16	286	**Sesaminol tetrahexoside I**
62	22.29	623.1981	624.2054	C_29_H_36_O_15_	0.37	0.23	98.18	461.1659, 315.1088, 297.0969, 179.0354, 161.0241, 153.0546, 135.0453, 113.0243	12	280, 308	Isoverbascoside
63	22.34	681.2389	682.2473	C_32_H_42_O_16_	2.13	1.45	94.88	519.1850, 357.1336, 327.0850, 297.0766, 161.0456, 151.0394, 149.0429, 137.0609	12	288	Pinoresinoldihexoside III
64	22.40	1017.3097	1018.3165	C_44_H_58_O_27_	−0.19	−0.19	99.14	855.2565, 693.2027, 369.0976, 323.0934, 221.0661, 219.0625, 179.0559, 161.0451, 149.0456	16	284	**Sesaminol tetrahexoside II**
65	22.56	841.2785	842.2857	C_38_H_50_O_21_	−1.42	−1.20	97.87	679.2245, 517.1755, 485.1514, 355.1189, 323.0984, 221.0668, 179.0563, 161.0459, 149.0455, 121.0295, 89.0244	14	285	**Xanthoxylol trihexoside**
66	22.93	163.0399	164.0473	C_9_H_8_O_3_	1.64	1.27	86.8	N.D.	6	284	*m*-Coumaric acid *
67	23.15	927.2789	928.2849	C_41_H_52_O_24_	−1.26	−1.17	97.47	883.2852, 841.2758, 823.2653, 679.2213, 661.2124, 485.1489, 467.1399, 355.1182, 323.0980, 221.0659, 161.0457, 179.0552, 177.0921, 149.0448, 121.0293	16	288	**Xanthoxylol malonyl trihexoside**
68	23.26	447.0942	448.1006	C_21_H_20_O_11_	−2.16	−0.97	85.48	N.D.	12	N.D.	Quercetin 3-*O*-rhamnopyranoside *
69	23.32	855.2585	856.2637	C_38_H_48_O_22_	−2.0	−1.7	96.4	693.2013, 485.1485, 369.0965, 323.0980, 221.0663, 219.0668, 179.0558, 161.0455, 149.0453, 143.0347, 119.0350	15	283	Sesaminol trihexoside I
70	23.45	855.2578	856.2637	C_38_H_48_O_22_	−1.8	−1.5	96.86	693.2329, 531.1494, 485.1508, 369.0978 221.0664, 219.0657, 179.0558, 161.0457, 149.0456, 143.0349, 119.0349	15	290	Sesaminol trihexoside II
71	24.25	871.2515	872.2586	C_38_H_48_O_23_	0.4	0.3	98.91	709.1974, 691.1869, 529.1376, 485.1509, 385.0926, 323.0985, 221.0665, 179.0556, 165.0192, 161.0457, 143.0347, 149.0452, 137.0243, 119.0349, 89.0243	15	292	**Hydroxysesamolin trihexoside**
72	24.32	531.1513	532.1581	C_26_H_28_O_12_	−0.7	−0.4	99.00	337.0929, 323.0776, 193.0503, 178.0267, 175.0398, 149.0607, 134.0370	13	292	**Diferuloyl hexoside**
73	24.45	317.0305	318.0388	C_15_H_10_O_8_	−3.7	−1.2	82.25	N.D.	11	N.D.	Myricetin *
74	26.45	285.0408	286.0477	C_15_H_10_O_6_	−1.2	−0.3	99.42	N.D.	11	288, 340	Luteolin *
75	26.65	301.0349	302.0420	C_15_H_10_O_7_	2.1	0.6	90.98	N.D.	11	N.D.	Quercetin *
76	27.01	593.1884 ^b^	594.1949	C_28_H_34_O_14_	−1.3	−0.8	98.05	371.1143, 356.0911, 233.0817, 161.0457, 138.0323	12	283	Sesamolinolhexoside
77	27.19	633.1829	634.1898	C_30_H_34_O_15_	−0.3	−0.2	99.43	N.D.	14	286	**Sesaminol dipentoside I**
78	27.43	633.1824	634.1898	C_30_H_34_O_15_	0.2	0.1	99.47	501.1317, 369.0969, 339.0969, 219.0646, 135.0341	14	285	**Sesaminol dipentoside II**
79	27.67	635.1984	636.2054	C_30_H_36_O_15_	−0.4	−0.2	98.82	371.1132	13	285	**Sesamolinol dipentoside I**
80	27.82	635.1991	636.2054	C_30_H_36_O_15_	−1.1	−0.7	96.65	371.1128	13	286	**Sesamolinol dipentoside II**
81	27.85	269.0457	270.0528	C_15_H_10_O_5_	−1.0	−0.3	83.23	N.D.	11	N.D.	Apigenin *
82	27.94	635.1980	636.2054	C_30_H_36_O_15_	0.2	0.1	99.43	371.1135	13	286	**Sesamolinol dipentoside III**
83	28.19	285.0403	286.0477	C_15_H_10_O_6_	0.7	0.0	84.77	N.D.	11	N.D.	Kaempferol *
84	28.32	271.0610	272.0685	C_15_H_12_O_5_	0.7	0.2	99.86	N.D.	10	N.D.	Naringenin *
85	29.81	255.0664	256.0736	C_15_H_12_O_4_	−0.23	−0.06	99.3	213.0551, 171.0440, 151.0026, 107.0136, 103.0534, 83.0134	10	288	Pinocembrin
86	29.84	299.0566	300.0634	C_16_H_12_O_6_	−0.6	−0.2	89.67	N.D.	11	N.D.	Kaempferide *

^a^ Detected ions were [M−H]^−^. ^b^ Detected ion was acetic acid adduct [M+CH_3_COOH − H]^−^. ^c^ Isomers are denoted with letter codes I, II, etc. * Identification confirmed by comparison with standards; N.D., below 5 mAU or masked by compound with higher signal. Compounds in bold letter indicate new proposed structures.

**Table 2:** Non-phenolic compounds characterized in the cake of the Egyptian cultivar of *Sesamum indicum* L. ‘Giza 32’.

Number	tR (min)	Experimental *m/z*^a^ [M − H]^−^	Theoretical Mass (M)	Molecular Formula	Error (ppm)	Error (mDa)	Score	Main Fragments	DBE	UV (nm)	Proposed Compound ^b^
1′	2.57	195.0513	196.0583	C_6_H_12_O_7_	−1.3	−0.3	99.39	135.0305	1	N.D.	Gluconic/Galactonic acid I
2′	2.59	131.0463	132.0535	C_4_H_8_N_2_O_3_	0.11	0.01	97.72	114.0110, 113.0358	2	N.D.	Asparagine
3′	2.65	195.0513	196.0583	C_6_H_12_O_7_	−0.9	−0.2	99.19	135.0297	1	N.D.	Gluconic/Galactonicacid II
4′	2.65	665.2144	666.2219	C_24_H_42_O_21_	0.54	0.36	98.67	503.1618, 341.1086, 179.0558	4	N.D.	Sesamose
5′	2.96	191.0198	192.027	C_6_H_8_O_7_	−0.2	−0	99.31	173.0087, 111.0089	3	N.D.	Citric acid I
6′	3.15	133.0143	134.0215	C_4_H_6_O_5_	−0.7	−0.1	99.61	115.0037	2	N.D.	Malic acid I
7′	3.45	133.0141	134.0215	C_4_H_6_O_5_	0.89	0.27	99.34	115.0039	2	N.D.	Malic acid II
8′	3.96	191.0197	192.027	C_6_H_8_O_7_	0.17	0.03	99.75	173.0086, 111.0088	3	N.D.	Citric acid II
9′	4.02	147.0296	148.0372	C_5_H_8_O_5_	1.3	0.19	97.42	103.0400	2	N.D.	Citramalic acid
10′	4.08	129.0191	130.0268	C_5_H_6_O_4_	1.9	0.25	99.4	85.0297	3	N.D.	Itaconic acid
11′	4.27	191.0203	192.027	C_6_H_8_O_7_	0.21	0.04	99.93	111.0086	3	N.D.	Citric acid III
12′	4.52	130.0872	131.0949	C_6_H_13_NO_2_	1.49	0.2	99.47	112.986	1	N.D.	Leucine/Isoleucine
13′	5.33	180.0663	181.0745	C_9_H_11_NO_3_	−1.7	−0.3	94.97	163.0406	5	264	Tyrosine *
14′	5.51	130.0874	131.0949	C_6_H_13_NO_2_	−0.4	−0.1	98.93	112.9860	1	N.D.	Leucine/Isoleucine
15′	6.29	611.1454	612.152	C_20_H_32_N_6_O_12_S_2_	−0.9	−0.6	98.87	481.1002, 338.0483, 306.0761, 288.0658, 254.0780, 179.0461, 128.0353	8	N.D.	Oxidized glutathione (Glutathione disulfide)
16′	7.00	171.0303	172.0372	C_7_H_8_O_5_	−3.4	−0.6	95.58	127.0403	4	230	(−)-3-Dehydroshikimic acid
17′	7.67	191.0569	192.0634	C_7_H_12_O_6_	−3.6	−0.7	96.92	147.0663, 129.0557, 101.0610	2	N.D.	Quinic acid I
18′	8.19	191.056	192.0634	C_7_H_12_O_6_	0.1	0.0	99.64	147.0655, 129.0551, 101.0604	2	N.D.	Quinic acid II
19′	9.12	164.0718	165.0790	C_9_H_11_NO_2_	−0.4	−0.1	99.53	147.0455, 129.0557, 85.0297	5	255	Phenylalanine *
20′	10.16	218.1034	219.1107	C_9_H_17_NO_5_	0.1	0.0	98.6	146.0819	2	N.D.	Pantothenic acid (Vit B5) I
21′	10.53	218.1038	219.1107	C_9_H_17_NO_5_	−1.4	−0.3	98.6	146.0822	2	N.D.	Pantothenic acid (Vit B5) II
22′	11.39	382.1003	383.1077	C_14_H_17_N_5_O_8_	−0.1	−0.1	98.1	266.0892, 250.574, 206.0679, 134.0468, 115.0034	9	265	Succinyladenosine
23′	12.72	529.1834	530.19	C_26_H_30_N_2_O_10_	−0.9	−0.5	99.2	203.0820, 159.0924, 142.0655, 116.0500	13	279, 287sh	Tryptophan derivative
24′	14.30	175.0611	176.0685	C_7_H_12_O_5_	0.62	0.11	99.62	115.04	2	N.D.	Isopropylmalic acid I
25′	14.61	175.061	176.0685	C_7_H_12_O_5_	1.41	0.25	99.21	115.04	2	N.D.	Isopropylmalic acid II
26′	24.03	187.0979	188.1049	C_9_H_16_O_4_	−1.8	−0.3	98.65	125.097	2	N.D.	Azelaic acid

^a^ Detected ions were [M − H]^−^. ^b^ Isomers are denoted with letter codes I, II, etc. * Identification confirmed by comparison with standards; N.D., below 5 mAU or masked by compound with higher signal.

## Supplementary Materials

The following are available online at https://www.mdpi.com/2304-8158/8/10/432/s1, Table S1: Phenolic compounds characterized in the cake of the Egyptian cultivar of sesame ‘Giza 32’, Table S2: Non-phenolic compounds characterized in the cake of the Egyptian cultivar of sesame ‘Giza 32’.
